# Transformational Leadership and Perceived Overqualification: A Career Development Perspective

**DOI:** 10.3389/fpsyg.2021.597821

**Published:** 2021-02-11

**Authors:** Man Zhang, Fan Wang, Haolin Weng, Ting Zhu, Huiyun Liu

**Affiliations:** ^1^Party School of Anhui Provincial Committee of C.P.C., Hefei, China; ^2^School of Management, Shanghai University, Shanghai, China

**Keywords:** transformational leadership, perceived overqualification, career growth opportunities, career development, career development perspective

## Abstract

Drawing on social information processing theory and a career development perspective, we examined the effect of transformational leadership on the perceived overqualification via career growth opportunities, and how the supervisor–subordinate guanxi moderates the relationship between transformational leadership and perceived overqualification. We tested this proposal using three waves of lagged data collected from 351 company employees in the Yangtze River Delta region in China. The results revealed that transformational leadership had an indirect effect on perceived overqualification through career growth opportunities, and supervisor–subordinate guanxi moderated the positive association between transformational leadership and career growth opportunities. In addition, the mediating effect of transformational leadership on perceived overqualification through career growth opportunities was stronger when the level of supervisor–subordinate guanxi was high and weaker when it was low. The findings have theoretical and practical implications for reducing employees’ perceptions of overqualification in the organizational context.

## Introduction

The concept of overqualification in the organizational literature has been described as an occupational condition in which individuals have more qualifications than necessary for the job (Erdogan and Bauer, 2009). Overqualification can be viewed objectively from the perspective of a potential employer comparing an individual’s qualifications with job requirements or subjectively from the perception of the employee or applicant. Objective overqualification is usually confirmed from the employer’s perspective by comparing the actual ability, education level and work qualification requirements of employees. The perception of employees’ occupation is an important factor affecting their attitude and behavior. In addition, objective overqualification is mainly researched in the field of sociology research. Therefore, scholars have started to focus on the influence mechanism of perceived overqualification, which refers to the degree to which individuals perceive themselves (or others) as having more qualifications than necessary for a job (Fine and Nevo, 2008). Historically, Empirical studies researchers focused on exploring the antecedents of perceived overqualification including individual factors (Erdogan and Bauer, 2020), such as narcissism (Maynard et al., 2015), age (Feldman, 1996; Feldman and Maynard, 2011; Liu and Wang, 2012), and gender (Feldman, 1996), and situational factors, such as objective overqualification (Kristof-Brown et al., 2005), job characteristics (Lobene et al., 2014), popularity (Li et al., 2019), and career adaptability (Yang et al., 2015). However, surprisingly little research has been done about the effects of leadership on an employee’s perceived overqualification, and we believe that more insight can be provided by investigating leader behaviors as an additional antecedent.

Employees’ perceptions of overqualification are influenced both by the job requirements and by organizational environmental factors (Hu et al., 2015). According to social information processing theory (Salancik and Pfeffer, 1978), individuals’ thoughts and behaviors are determined not only by their needs and goals but also by environmental cues. The interpretation of this environmental information determines the individual’s subsequent attitudes (Salancik and Pfeffer, 1978). Leaders, as important agents of the organizational environment, have a crucial influence on followers (Hussain et al., 2017; Bavik et al., 2018; Lee et al., 2020). Previous studies have examined how leaders influence the attitudes of their subordinates through their leadership style (Avolio and Bass, 1991; Bass and Avolio, 1999), in particular through transformational leadership behaviors. Transformational leaders appeal to the ideals and morals of their followers to inspire the followers to reach their highest levels of achievement and make employees aware of the importance of their tasks (Burns, 1978; Bommer et al., 2005; Farahnak et al., 2019). Under transformational leader, employees value the interests of the organization more than their own, and may increase incremental contributions through exerting effort beyond the call of duty as a response to the motivational signals of transformational leadership. the literature on transformational leadership has demonstrated its associated with improved staff attitudes, such as job satisfaction (Walumbwa et al., 2005) and organizational commitment (Bycio et al., 1995), as well as decreased negative outcomes, such as turnover intentions (Bycio et al., 1995) and burnout (Corrigan et al., 2002). However, research on the relationship between transformational leadership and employees’ perceived overqualification remains limited. As a result, it is important to consider whether transformational leadership as an antecedent affects employee’s perceived overqualification. This is the first issue we explored in this study.

Previous studies have shown that perceived overqualification not only refers to a perception formed by individuals when comparing their qualifications with job requirements but also to the perception of their current status in the development of their career (Newland, 2017). From a career development perspective, when choosing a career, employees mainly focus on whether the organization can provide them with opportunities for career growth and development, namely career growth opportunities (Weng et al., 2010). Therefore, it is reasonable to believe that career growth opportunities play a key role in shaping people’s perceived overqualification. In addition, leaders, as a key situational factor, are an important foundation for the career development of subordinates and have a significant effect on their career growth opportunities (Crawshaw and Game, 2014). Sosik et al. (2004) proposed that transformational leadership positively affects the career growth of subordinates by motivating their learning goals. Furthermore, transformational leadership focuses on creating an atmosphere of learning and innovation and encourages employees to make full use of their qualifications and potential, thereby enabling them to increase their career growth expectations. In summary, employees view the information from the compelling vision of the future and incentives to challenge the *status quo* provided by transformational leadership as the foundations of career growth opportunities (Bass, 1985; Berson and Avolio, 2004). Career growth opportunities in turn provide important cues that inform employees about the nature and development of the work, including whether employees have more qualifications than necessary for the current and future job. Accordingly, it is important to apply social information processing theory to determine whether career growth opportunities play a mediating role in the relationship between transformational leadership and perceived overqualification. This is the second issue we explored in this study.

**FIGURE 1:**
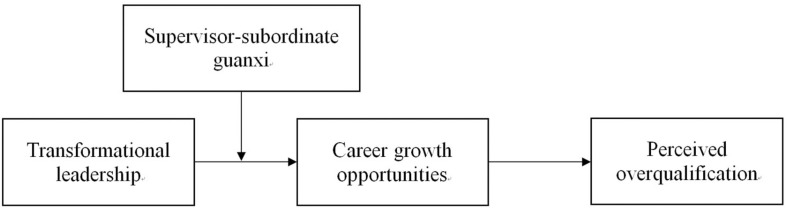
Theoretical model.

The influence of leadership style on employees’ career development may not always exist and may be affected by situational factors. Therefore, we posited that there are boundary conditions for the relationship between transformational leadership and employees’ career growth opportunities. Recent research in China has shown that guanxi is an important variable explaining individual outcomes in Chinese firms (Law et al., 2000; Chen and Tjosvold, 2007; Bruton and Lau, 2008). Guanxi is an indigenous Chinese construct describing an informal connection between two or more individuals or groups involving shared social experience and the exchange of favors and trust (Bian and Ang, 1997). Supervisor–subordinate guanxi is the most important interpersonal relationship in various Chinese organizational contexts (Law et al., 2000; Bruton and Lau, 2008; Chen et al., 2013). Unlike leader-member exchange (LMX), which reflects the quality of exchange between the supervisor and the subordinate in the workplace only, supervisor–subordinate guanxi can be accumulated through non-work-related activities, such as dinners, gift giving, and doing favors (Law et al., 2000). It involves group cognition and social emotional elements that go beyond the work relationship (Wei et al., 2010). Previous research has shown that supervisor–subordinate guanxi plays a key role in the career success of subordinates (Wei et al., 2010). Therefore, we predicted that transformational leadership can more effectively improve employees’ career growth opportunities when the level of supervisor–subordinate guanxi is high. In other words, supervisor–subordinate guanxi moderates the relationship between transformational leadership and career growth opportunities. To test our predictions, drawing on social information processing theory and a career development perspective, we constructed a moderated mediation model to explore the relationship between transformational leadership and perceived overqualification. The graphical representation of the proposed model is provided in Figure 1.

This study contributes to the literature in several ways. First, some studies have explored the effects of perceived overqualification, such as low job satisfaction, low psychological well-being, and high turnover intention (Johnson and Johnson, 1996; Johnson et al., 2002; Erdogan and Bauer, 2009; Luksyte et al., 2011; Liu et al., 2014). However, a frequently mentioned but unexplored problem in the literature is the lack of analysis of the antecedents of perceived overqualification. We addressed this research gap by examining the effect of transformational leadership on perceived overqualification. Second, based on social information processing theory, this study attempted to explain the mediating role of career growth opportunities, which will help to explain the mechanism by which transformational leadership affects perceived overqualification, offering a new perspective and new ideas for future research. Finally, previous research has identified individual characteristics (Aryee et al., 2007) or contextual factors (Mawritz et al., 2012) that limit the career development of employees. Yet, few studies have examined the effect of guanxi between leaders and employees on career development processes. This study addressed this question by introducing supervisor–subordinate guanxi as a situational variable, enriching the research on the boundary conditions of the antecedents of overqualification and providing another conditional mechanism for leadership effectiveness in the context of China.

## Theory and Hypotheses

### Transformational Leadership and Perceived Overqualification

According to social information processing theory, the social environment in which individuals live provides a variety of information, and their interpretation of this information determines their subsequent attitudes and behavior (Salancik and Pfeffer, 1978). As an important source of information in the work environment, leaders’ thoughts, attitudes, and behavior are the basis of their employees’ cognition, attitudes, and behavior (Baruch, 2004, 2006; Sturges et al., 2010). As the information sent by leaders in the work environment can influence employees only if they process the information, social information processing theory is the basic theory needed to explain the downward transmission effect of leadership behavior. Therefore, this study draws on social information processing theory to explore the relationship between transformational leadership and employees’ perceptions of overqualification and its mechanism.

First, according to social information processing theory, leadership, as the main situational factor in the organizational environment, can shape employees’ cognition and affect their psychology and behavior (Salancik and Pfeffer, 1978). Transformational leadership is an effective leadership style that focuses on stimulating employees’ potential by having high expectations for them and constantly encouraging them to challenge themselves to achieve better goals. This process provides employees with targeted care and training, including the opportunities, support, respect, and resources needed for development, to motivate them to achieve results beyond expectations. Transformational leadership can effectively convey a positive vision of the future of the organization, which can attract employees’ attention, improve their work motivation, and empower them to transform their overqualification into implementation strategies to realize this vision (Avolio and Bass, 1991; Bass and Avolio, 1999). In this case, overqualified employees will find a balance between their current position and the future development of the organization. Second, transformational leadership tends to enhance employees’ work expectations and encourages them to strive for excellence by giving meaning to work (Burns, 1978). In this case, there is reason to believe that overqualified employees will be motivated to take on new challenges in their career development and internalize these tasks into opportunities for career success (Ng and Feldman, 2010), thereby reducing their feeling of overqualification. Third, transformational leadership emphasizes innovation and learning (Pieterse et al., 2009), in which employees can make full use of their talents and qualifications. Therefore, overqualified employees are more likely to feel valued and to develop. In addition, implementing personalized care strategies through transformational leadership enables leaders to care about the individual needs of each member of the organization, discover the potential of each member, and guide them to accomplish their tasks. Consequently, transformational leaders will fully consider the unique characteristics of overqualified employees and provide different types of support and continuous skill development.

Thus, based on the reasoning and literature discussed above, we proposed the following.

Hypothesis 1: Transformational leadership is negatively associated with employees’ perceived overqualification.

### Mediating Effect of Career Growth Opportunities

The concept of career growth has been studied extensively by scholars in the fields of psychology, human resources, and sociology (Adamson et al., 1998). Research has defined career growth as acquiring better skills, more responsibility and prestige, and higher income through a series of jobs, including carrying out increasingly more advanced or diversified activities (Bloomsbury Business, and Management Dictionary., 2007). Career growth opportunities refer to the opportunities for better career development offered by employers to increase the relevant knowledge and skills of their employees, such as taking on more challenging tasks, taking on more responsibility, and gaining more relevant experience (Weer et al., 2006). When making career choices, employees not only pursue material and spiritual needs but also consider their career development needs. This is a key condition for employees to learn, train, and grow in their organization.

Based on the theory of social information processing, leadership style can shape employees’ cognition and affect their psychology and behavior (Salancik and Pfeffer, 1978). Transformational leadership focuses on long-term interests, motivates employees’ potential, provides development opportunities and support for employees, and improves employees’ expectation of results. Some studies have shown that outcome expectations might have an important influence on employees (Pham et al., 2020). Thus, this study proposes that transformational leadership can improve the career growth opportunities of employees. First, transformational leadership can improve employees’ enthusiasm and initiative by increasing their responsibility and mission, in turn improving their opportunities for career development. Second, transformational leadership focuses on strengthening cooperation and communication between teams and providing employees with more development space and career growth opportunities (Scott and Caress, 2005). In this case, employees will benefit from the support, encouragement, and trust of their leaders, thereby increasing their career development opportunities. Third, developing personalized transformational leadership strategies encourages leaders to focus on the needs of each member of the organization and provide different types of support and continuous skill development, increasing their career growth opportunities. Therefore, we believe that in organizations using transformational leadership, employees are more likely to obtain career growth opportunities. Studying the effect of transformational leadership mechanisms, Ng (2017) made it clear that transformational leaders can ask personalized questions and provide personalized support to subordinates, to help them realize their professional and personal goals.

Furthermore, according to social information processing theory, career development opportunities—as an outcome of the expectations of employees engaged in a particular occupation—can affect the assessment of their qualifications (Maltarich et al., 2011). When employees have good career growth opportunities, they are more comfortable in their jobs, which is good for the performance of the entire organization (Schaufeli and Bakker, 2004). Moreover, career growth opportunities help overqualified employees to be more satisfied with their work and organization (Kahn, 1990), so they are likely to take the initiative to make full use of their qualifications and potential to pursue higher work goals, helping them reduce their feeling of overqualification.

In summary, transformational leadership can enhance the career growth opportunities of employees and reduce their feeling of overqualification. Therefore, we proposed the following hypotheses.

Hypothesis 2: Career growth opportunities are negatively associated with employees’ perceived overqualification.
Hypothesis 3: Career growth opportunities mediate the relationship between transformational leadership and employees’ perceived overqualification.

### Moderating Role of Supervisor–Subordinate Guanxi

Previous studies have shown that transformational leadership is effective in organizations (Bass et al., 2003; Carter et al., 2012). However, in the context of Chinese organizations, the influence of leader behaviors on employees cannot be separated from guanxi. Guanxi is a key concept for understanding Chinese social structure and interpersonal communication, and refers to the connection between different parties and an extended network of interpersonal relationships involving the exchange of favors (Bian and Ang, 1997; Farh et al., 1998). Guanxi is often translated into English as “relationships,” but the concept differs in that relationships can be positive or negative, while guanxi can be only strong or weak. Guanxi was the basis of social order and hierarchy in ancient China and is still important today. Although the structure and nature of guanxi have evolved, modern Chinese societies remain focused on guanxi (Chen and Chen, 2004). Supervisor–subordinate guanxi refers to the interpersonal relationship between supervisors and subordinates (Wei et al., 2010). Unlike LMX, supervisor–subordinate guanxi can be accumulated through non-work related social activities (such as dinners, gift giving, and doing favors), but it affects interactions at work (Law et al., 2000). Supervisor–subordinate guanxi involves group cognition and social emotional elements that go beyond the work relationship (Wei et al., 2010). As supervisors are essential organizational resources and directly determine the performance assessment, promotion, and empowerment of their subordinates (Wei et al., 2010), supervisor–subordinate guanxi is instrumental in the effective implementation of transformational leadership and the provision of career growth opportunities for employees (Wei et al., 2010). Based on this, this study examined whether supervisor–subordinate guanxi plays a moderating role in the relationship between transformational leadership and career growth opportunities.

In Chinese enterprises, higher levels of supervisor–subordinate guanxi indicate that subordinates have become “insiders” of leaders (Law et al., 2000). Previous studies have shown that leaders adopt different management methods and strategies in terms of emotional attachment and resource allocation between subordinates according to the status of employees “inside” and “outside” the circle. In other words, by using transformational leadership to implement strategies such as intellectual stimulation, the “insider” status can provide employees with more opportunities for skills development, so that their unique needs and potential can be better realized. With the help of these resources and care, they will have more career growth opportunities. Furthermore, based on the principle of mutual beneficial social exchange, high levels of supervisor–subordinate guanxi can create a relationship of trust between the two parties (Neubert et al., 2013). On the one hand, the leadership behavior of leaders is more likely to gain support and understanding from their subordinates; on the other hand, employees are likely to support their leaders and engage in better work behavior (Farh et al., 2007), thereby helping them to gain more support and encouragement at work and increase their career growth opportunities. Specifically, when the level of supervisor–subordinate guanxi is high, the positive relationship between transformational leadership and career growth opportunities is stronger.

Conversely, lower levels of supervisor–subordinate guanxi mean that the emotional attachment between leaders and subordinates is not established. When leaders carry out their work, “outside” employees may not support and cooperate with them, especially overqualified employees who feel that their abilities are not valued, leading to misunderstandings and dissatisfaction with leaders. Furthermore, leaders may pay less attention to employees with weaker supervisor–subordinate guanxi, thus “outside” employees have fewer opportunities to demonstrate their experience and skills, hindering their potential career growth opportunities. In summary, we proposed the following moderating effect of supervisor–subordinate guanxi.

Hypothesis 4: Supervisor–subordinate guanxi moderates the relationship between transformational leadership and career growth opportunities, such that the positive effect is stronger when the level of supervisor–subordinate guanxi is high than when it is low.

Hypotheses 1 to 4 present the relationships constituting the overall moderated mediation model. This study further examined whether supervisor–subordinate guanxi not only moderates the relationship between transformational leadership and career growth opportunities but also moderates the indirect effect of transformational leadership on employees’ perceived overqualification through career growth opportunities. On this basis, we proposed the following hypothesis.

Hypothesis 5: Supervisor–subordinate guanxi moderates the relationship between transformational leadership and perceived overqualification through career growth opportunities, such that the indirect effect is stronger when the level of supervisor–subordinate guanxi is high than when it is low.

## Materials and Methods

### Participants and Procedure

Two companies from the Yangtze River Delta region were recruited to participate in a field survey. The first company was a large motor company headquartered in Shanghai, China, and the other company was a well-known enterprise in China’s heavy equipment manufacturing industry, headquartered in Shanghai. Through preliminary interviews and with the help of the human resource managers of the two firms, we found that the recruitment requirements of the two companies, such as education level, were relatively high in the industry, but compared with the overall industry level, their salary level did not have a clear competitive advantage. These characteristics made the participating companies suitable for our research. To reduce common method variance and illusory correlations, we collected data in three waves between June and October 2018.

Due to the uncertainty of employee access to computers, to ensure the amount of data we collect, we use paper-based surveys to gather data in time 1. We invited 500 subordinates who were divided into several waves to fill the questionnaire in the meeting rooms and then issued questionnaires through the one-to-one correspondence way between questionnaire number and employees’ work number. We also explained the purpose of the study, emphasizing that data was only used for scientific research. More importantly, we encourage participants to provide a valid e-mail address or WeChat ID (the popular Chinese mobile messenger app) and actively participate in the second research online. At the end of the survey, we gave each participant about $5 in cash and promised that we will give $10 for the end of our surveys.

In order to improve our research efficiency, we used an online survey website 1 month later (Time 2). With the help of Wenjuanxing website^1^, the Chinese version of Qualtrics, each questionnaire had a unique questionnaire ID automatically generated within Wenjuanxing. Based on the collected information of the paper version of the questionnaire, we made one-to-one correspondence between the questionnaire number, work number, email address and the Wenjuanxing ID and recorded them. We then sent the second questionnaire link to the 418 subordinates who provided a valid email address or WeChat IDs. At Time 2, 387 participants completed the second-stage survey. One month later (Time 3), these 387 participants were asked to complete the third-stage questionnaire on perceived overqualification using the same channel as T2. Finally, 351 participants from 68 groups completed the three stages of the survey, for a total response rate of 70.2%. Each group consisted of three to six employees. Of the 351 employees, 81.48% were men, 86.33% were aged 39 or younger, 56.41% had a Bachelor’s degree, and the mean organizational tenure was 1.68 years. Further, no significant difference was identified in a *t*-test between demographics for all employees, which were collected from both T1 and T3, and checks for non-response bias were conducted. There were no differences between invitees who did and did not respond in terms of gender (*t* = 0.71, *p* > 0.05), and perceived overqualification (*t* = 0.62, *p* > 0.05). Hence, we could guarantee that all surveys listed were matched across time.

### Measures

All measures were in Chinese and followed the standard translation–back translation process (Brislin, 1986). The participants completed the measures using a 5-point Likert scale, unless otherwise noted (1 = *strongly disagree* to 5 = *strongly agree*).

#### Transformational Leadership

Transformational leadership was measured at Time 1 using the 6-item scale developed by Schippers et al. (2008), widely used by previous researchers (De Hoogh et al., 2005; Lin et al., 2019). Sample items include “My leader serves as a role model for me” and “My leader encourages me to look at problems from new angles.” Cronbach’s alpha for the scale was 0.900.

#### Supervisor–Subordinate Guanxi

We asked the participants to rate the level of guanxi with their supervisors at Time 2. Supervisor–subordinate guanxi was measured using the 6-item scale developed by Law et al. (2000), which has been shown to be suitable for work situations in China (Warren et al., 2004). Sample items include “I will invite leaders to lunch/dinner” and “I always actively share my personal thoughts, problems, needs, and feelings with my supervisor.” Cronbach’s alpha for the scale was 0.894.

#### Career Growth Opportunities

We used the 4-item career growth opportunities scale developed by Bedeian et al. (1991). The scale was rated by the participants at Time 2 to measure the expected utility of their current work in achieving their career outcomes. Sample items are “I think my present job can help me achieve my future career goals” and “My current job is related to career growth.” Cronbach’s alpha for the scale was 0.902.

#### Perceived Overqualification

We used the 9-item perceived overqualification scale developed by Maynard et al. (2006). The scale measures employees’ perceived overqualification in terms of knowledge, skills, abilities, and work experience. A sample item is “Even though I don’t have previous work experience, I can successfully complete my current job.” Cronbach’s alpha for the scale was 0.846.

#### Control Variables

Previous empirical studies have confirmed that narcissism is positively related to individuals’ perceived overqualification, due to people’s strong narcissistic tendency to exaggerate and emphasize their ability (Maynard et al., 2015; Harari et al., 2017). Thus, we controlled for narcissism in our analysis. We used the 9-item measure of narcissism developed by Jones and Paulhus (2014), which has been validated and used in other studies (Maynard et al., 2015; Harari et al., 2017). Rated on a 5-point Likert-type scale (1 = *strongly disagree* to 5 = *strongly agree*), sample items are “People with perceived overqualification will insist on being treated with respect” and “You know you’re different because everyone says so.” Cronbach’s alpha for this scale was 0.820. The level of education has also been shown to be an important antecedent of overqualification (Hung, 2008). Therefore, we controlled for the education level, gender, and age of the participants. We further controlled for the job tenure of both leaders and subordinates because it takes some time to establish a supervisor–subordinate relationship.

### Method of Analysis

Although all of the variables in our proposed theoretical model were measured at the individual level, our data were nested because employees on multiple teams evaluated the transformational leadership style of co-leaders. To account for these nested effects, we adopted the multilevel path analysis approach (Preacher et al., 2010, 2016; Hamaker and Muthén, 2020) using Mplus 7.4 (Muthén and Muthén, 1998-2016) to test the proposed hypotheses. The multilevel structural equation model (MSEM) can not only deal with latent and explicit variables simultaneously, thus eliminating the measurement error of dominant variables, but also provides a complete model to fully explain the direct and indirect relationships. Following the recommendations of Preacher and Selig (2012), the confidence intervals (CIs) of the high and low standard deviation groups reporting indirect effects were calculated using Monte Carlo parameter sampling to estimate the 95% CIs and determine their significance.

## Results

### Confirmatory Factor Analysis (CFA)

We conducted a multilevel CFA to explore and distinguish between the substantive constructs in the study. Bandalos (2002) argued that the inclusion of all measurement items as observed indicators in the original model will result in parameter estimation bias, as the recommended parameters to sample size ratio will be exceeded. As the perceived overqualification scale has been used as a unidimensional scale in the literature, we followed the recommendations of Arvan et al. (2019) and created 3-item parcels for each dimension of the scale: overeducation, excess knowledge, skills, and over experience. The score for each parcel was calculated by taking the average score of the three items assigned to it. The results of our multilevel CFA, shown in Table 1, indicated that our proposed four-factor (transformational leadership, supervisor–subordinate guanxi, career growth opportunities, and perceived overqualification) within-group and between-group models fit the data well [χ*^2^* = 545.078, *df* = 300, RMSEA = 0.048, TLI = 0.926, CFI = 0.935, SRMR = 0.082 (within-group level) and 0.481 (between-group level)]. All items loaded significantly on their corresponding factors. These models provided a better fit to the data than all six constrained models in which any two of the six factors at the individual level were combined, and all six constrained models in which any two of the four factors at the group level were combined (Appelbaum et al., 2018). These results confirmed the dimensionality and discriminant validity of our measures at both the within- and between-group levels.

**TABLE 1:** Model fit results for multilevel confirmatory factor analyses and conventional confirmatory factor analyses.

Model	χ2	*df*	CFI	TLI	RMSEA	SRMR		AIC	BIC
						Within	Between		
**Conventional CFA Models**									
Model 1	270.536	146	0.968	0.963	0.049	0.037		16,608.290	16,851.519
Model 2	599.337	149	0.884	0.867	0.093	0.076		17,160.131	17,391.778
Model 3	1,657.920	151	0.613	0.561	0.169	0.123		17,789.685	18,013.611
Model 4	1,975.430	152	0.531	0.473	0.185	0.137		18,301.183	18,521.248
**Multilevel CFA Models**									
Model 5	545.078	300	0.935	0.926	0.048	0.082	0.481	16,757.264	17,139.481
Model 6	571.348	302	0.876	0.852	0.096	0.093	0.462	17,768.458	17,686.137
Model 7	2,071.205	306	0.0531	0.476	0.128	0.162	0.369	18,271.391	18,630.444

*df, degrees of freedom; CFI, comparative fit index; TLI, Tucker–Lewis index; RMSEA, root mean square error of approximation; SRMR, standardized root means square residual; AIC, Akaike information criterion; BIC, Bayesian information criterion. Model 1, hypothesized four-factor model; Model 2, three-factor mode (perceived overqualification and supervisor–subordinate guanxi are combined); Model 3, two-factor mode (perceived overqualification, supervisor–subordinate guanxi, and Career growth opportunities are combined); Model 4, single-factor model (all four variables are combined); Model 5, four-factor (within) and four-factor (between); Model 6, three-factor (within) and three-factor (between) (perceived overqualification and supervisor–subordinate guanxi are combined); Model 7, one-factor (within) and one-factor (between). We chose the best-fitting three-factor and two-factor models.*

Conventional confirmatory factor analysis was also used to assess the distinctiveness of the four variables used in the test model: transformational leadership, supervisor–subordinate guanxi, career growth opportunities, and perceived overqualification. Four models with different configurations of these four variables were tested, and the final CFA results showed that the fit indices for the four-factor model were better than those of the other nested models (χ*^2^* = 270.536, *df* = 146, RMSEA = 0.049, TLI = 0.963, CFI = 0.968). Therefore, all of the individual variables had acceptable discriminant validity.

### Descriptive Statistics and Correlations

The means, standard deviations, and correlations for each variable studied are provided in Table 2. The results showed that transformational leadership was significantly correlated with career growth opportunities at the individual level (*r* = 0.325, *p* < 0.01). As expected, career growth opportunities were negatively related to perceived overqualification at the individual level (*r* = −0.226, *p* < 0.01). Thus, the correlation results were in line with theoretical expectations and provided a basis for further analysis.

**TABLE 2:** Means, standard deviations, and correlations.

Variables	*M*	*SD*	1	2	3	4	5	6	7	8	9
1. Gender	1.180	0.387									
2. Age	34.336	6.655	−0.147*								
3. Education	3.260	0.777	−0.186**	0.130*							
4. Job tenure	5.760	3.6632	–0.076	0.295**	0.034						
5. Narcissism	2.524	0.501	–0.053	–0.078	–0.053	–0.010	(0.978)				
6. TL	4.014	0.878	–0.009	–0.073	–0.008	−0.142**	−0.1086*	(0.900)	0.266**	0.650**	−0.362**
7. CGO	2.855	1.229	0.015	−0.116*	–0.014	–0.064	–0.068	0.325**	(0.902)	0.273**	−0.373**
8. SSG	3.718	0.863	–0.021	–0.055	0.002	–0.070	–0.058	0.538**	0.266**	(0.847)	−0.378**
9. POQ	2.969	0.848	0.005	–0.078	0.203**	–0.047	0.205**	−0.165**	−0.226**	−0.217**	(0.846)

For all variables, Cronbach’s alpha values are reported in brackets along the diagonal. TL, transformational leadership; CGO, career growth opportunities; SSG, supervisor–subordinate guanxi.*p < 0.05, **p < 0.01. The correlations above the diagonal represent between-level correlations (computed using individuals’ aggregated scores; n = 68). The correlations below the diagonal represent within-level correlations (n = 351).

### Hypothesis Testing

The results of analysis of variance (ANOVA) show significant variances at the group level for perceived overqualification, *F*(67,351) = 1.931, *p* < 0.001. Besides, the estimated ICC (1) = 0.151, implying that 15.1% variances of perceived overqualification stem from the group level factors. The estimated ICC (1)s are 0.198 for transformational leadership and 0.142 for supervisor–subordinate guanxi and 0.032 for career growth opportunities, implying that around 19.8% variances of transformational leadership and 14.2% variances of supervisor–subordinate guanxi and 3.2% variances of career growth opportunities were attributable to the group level factors.

**FIGURE 2:**
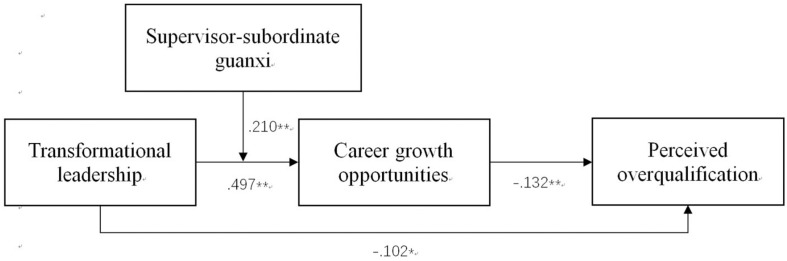
Multilevel SEM results. ^∗^*p* < 0.050, ^∗∗^*p* < 0.010.

**FIGURE 3:**
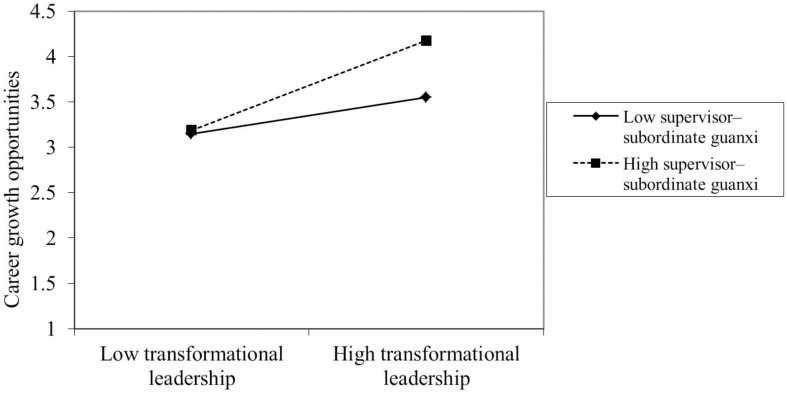
The effect of the interaction between transformational leadership and supervisor–subordinate guanxi on career growth opportunities.

After controlling for the influence of the control variables, the results of the MSEM analysis are summarized at the bottom of Figure 2. The detailed results are presented in Table 3. The path coefficient showed a positive correlation between transformational leadership and career growth opportunities (γ = 0.497, *p* < 0.01). Career growth opportunities had a negative effect on perceived overqualification based on the path coefficient (γ = −0.132, *p* < 0.01), after ruling out the between-level variance and controlling for the participants’ education level and job tenure. In addition, the path coefficient for transformational leadership was negatively correlated with the direct effect of perceived overqualification (γ = 0.102, *p* < 0.05). Thus, Hypothesis 1 and Hypothesis 2 were supported. Moreover, the relationship between transformational leadership and career growth opportunities was moderated by supervisor–subordinate guanxi (γ = 0.210, *p* < 0.01). Figure 3 and a separate simple slope analysis further revealed that the positive relationship between transformational leadership and career growth opportunities was stronger for people with high levels of supervisor–subordinate guanxi (γ = 0.622, SE = 0.123, *t* = 5.071, p = 0.000) than low levels (γ = 0.272, SE = 0.097, *t* = 2.808, *p* = 0.000). Thus, Hypothesis 4 was supported.

**TABLE 3:** Results of multilevel path analyses.

Outcome variable:	POQ		CGO
	M1	M2	M3
Intercept (γ_00_)	2.723**	2.405**	1.652**
Age (γ_20_)	**−**0.010	**−**0.013*	0.023*
Gender (γ_30_)	0.010	0.081	0.418**
Education (γ_40_)	0.058**	0.022**	0.193*
Job tenure (γ_50_)	**−**0.013	**−**0.014	0.007
Narcissism (γ_60_)	0.231**	0.309**	0.341**
TL (γ_10_)	**−**0.102*	0.06	0.497**
SSG (γ_01_)			0.239**
TL × SSG (γ11)			0.210**
CGO (γ_7__0_)		**−**0.132**	
POQ (γ_8__0_)			
σ^2^	0.572	0.564	0.145
τ_00_	0.109	0.004	0.272
AIC	9,401.964	3,746.685	3,912.798
BIC	9,552.535	3,823.901	3,986.153
Pseudo *R*^2^	0.059	0.072	0.158

*^∗^p < 0.05, ^∗∗^p < 0.01.POQ, perceived overqualification; CGO, career growth opportunities; TL, transformational leadership; SSG, supervisor–subordinate guanxi. σ^2^, within-level residual; τ_00_, between-level residual. Pseudo R^2^ indicates the proportion of variance explained in the dependent variable after the research model variable is entered into the regression equation. See the previous explanation for calculation (Snijders and Bosker, 1994).*

As Table 4 shows, the mediating effect of career growth opportunities on the relationship between transformational leadership and perceived overqualification was confirmed [γ = −0.165, SE = 0.188, 95% CI (−0.100, −0.031), excluding 0]. Therefore, Hypothesis 3 was supported.

**TABLE 4:** Mediating effect of career growth opportunities on the relationship between transformational leadership and perceived overqualification.

	γ	SE	95% confidence interval	
			Lower limit	Upper limit
Within-group mediating effect	−0.066	0.018	−0.100	–0.031
Between-group mediating effect	−0.165	0.188	−0.533	0.203

As Table 5 shows, when the level of supervisor–subordinate guanxi was low, the relationship between transformational leadership and perceived overqualification through career growth opportunities was significant and negative [γ = −0.084, 95% CI (−0.065, −0.006), excluding 0]. Correspondingly, when the level of supervisor–subordinate guanxi was high, the relationship between transformational leadership and perceived overqualification through career growth opportunities was still negative, but the relationship was weaker [γ = −0.036, 95% CI (−0.139, −0.028), excluding 0]. The difference between the two levels was significant, with 95% CI (−0.090, −0.006), excluding 0. Therefore, Hypothesis 5 was supported.

**TABLE 5:** Results of the moderated mediation effect between transformational leadership and perceived overqualification.

Group statistics	γ	SE	95% CI	
			Lower limit	Upper limit
Conditional indirect effect				
High supervisor–subordinate guanxi (+1 SD)	−0.085	0.029	−0.141	−0.029
Low supervisor–subordinate guanxi (−1 SD)	−0.036	0.014	−0.064	−0.008
DIFF	−0.049	0.022	−0.093	−0.006

## Discussion

Integrating social information processing theory and a career development perspective, our study developed a theoretical model of transformational leadership and perceived overqualification. We used transformational leadership as the antecedent of perceived overqualification and introduced career growth opportunities to explain the influence mechanism through which transformational leadership affected perceived overqualification, from the new perspective of career development. In addition, our study added supervisor–subordinate guanxi as a boundary condition. Based on a sample of 351 participants, the results revealed that transformational leadership was negatively related to perceived overqualification through career growth opportunities, and that supervisor–subordinate guanxi moderated the positive relationship between transformational leadership and career growth opportunities, such that the relationship was stronger for individuals with higher levels of supervisor–subordinate guanxi. We also found that the indirect effect of transformational leadership on perceived overqualification was moderated by supervisor–subordinate guanxi.

### Theoretical Implications

Our study makes several contributions to the literature. First, based on social information processing theory, we highlight how transformational leadership affects perceived overqualification. In other words, this study fills an existing research gap. In addition, despite a large body of theoretical studies on specific forms of perceived overqualification, little empirical research has explored the influence mechanism of antecedent variables on perceived overqualification. Responding to the call to explore the antecedent mechanism of perceived overqualification, using a career development perspective, our results suggest that transformational leadership can improve employees’ career growth opportunities and help reduce their feeling of overqualification. Therefore, we expand the literature on perceived overqualification by identifying a new interpersonal antecedent, namely transformational leadership.

Second, this study introduces career growth opportunities and identifies it as a mediator between transformational leadership and perceived overqualification. Prior researchers have called for additional studies to explore potential mediators of perceived overqualification to better understand its antecedents. Based on social information processing theory, this study shows that transformational leadership can improve employees’ outcome expectations and enhance their career growth opportunities (Shahibudin, 2015). Career growth opportunities affect employees’ cognitive judgment of their qualifications. Therefore, this finding indicates that the effect of transformational leadership on perceived overqualification can be interpreted as a career development process whereby employees can enhance their career identification and reduce their perceived overqualification. In doing so, we expand research of the association between transformational leadership and perceived overqualification. Furthermore, using a career development perspective, our results highlight the negative effect of transformational leadership and career growth opportunities on perceived overqualification. Taken together, this study deepens the understanding of the effective process of transformational leadership and provides a new perspective and new ideas for future research.

Third, this study uses supervisor–subordinate guanxi as a boundary condition to explain the influence of transformational leadership on perceived overqualification. Deng et al. (2016) first proposed a relational perspective to explore the interpersonal influence of overqualified individuals on different types of social acceptance, which in turn affects their in-role performance and their positive work-related behaviors. However, their research focused on relationships with co-workers and neglected relationships with direct leaders, which have been seen as the most important interpersonal relationships at work. Our study extends the work of Deng et al. (2016) by exploring the effect of interpersonal relationships (relationships with employees and supervisors) in the context of overqualification. As expected, we found that supervisor–subordinate guanxi moderated the mediating effect of transformational leaders on perceived overqualification through career growth opportunities. Notably, our study focused on supervisor–subordinate guanxi, while previous studies have paid more attention to leader–member exchange relationships, which are limited to the workplace. According to Law et al. (2000), examining supervisor–subordinate guanxi through substantive behaviors such as private visits, gift giving, and greetings can not only accurately measure the level of guanxi but also provide an additional conditional mechanism for leadership effectiveness in the Chinese context.

### Practical Implications

Overall, this study provides insight into organizational management and human resource practices. First, with the expansion of higher education, the phenomenon of overqualification has become common in organizations, and it is inevitable that some employees will feel overqualified. Therefore, leaders should actively encourage these employees to use their underutilized resources to fulfill their core responsibilities while succeeding in other areas of performance that require outstanding qualifications, such as creative performance. It is important for organizations and leaders to understand that although overqualified employees may be a risk factor, if managed properly, they can be an asset to the organization (Erdogan et al., 2011).

Second, in terms of organizational management practices, especially in the development of teamwork, this study provides insight into the interaction between employees and leaders. The leadership style of leaders has an important influence on the work attitudes and behaviors of employees, and an excellent leadership style plays a key role in improving the performance of organizations and employees. Therefore, for leaders, the choice of leadership style should be based on their current social environment and the growth stage of their organization. When decision makers and business professionals are faced with decision making, they should fully consider the personal qualifications of employees and give them the appropriate authority to improve their enthusiasm at work. In addition, decision makers and business professionals should formulate appropriate policies and ensure smooth promotion channels to improve employees’ sense of identity with the organization and increase their work engagement.

Finally, for employees, when making career choices, they generally pay special attention to their potential growth and development. Previous studies have shown that both in the West and in the Chinese context, career growth opportunities in the psychological contract of employees carry a very high weight (Rousseau, 1990), especially for employees pursuing higher career goals. The basis for an organization to attract high-quality employees is to train and harness the potential of its employees and to provide them with smooth promotion channels. In addition, an organization should provide opportunities for its employees to grow, to retain talent and reduce employee turnover. This can also help to reduce employees’ perceived overqualification and thus improve the performance of the whole team.

### Limitations and Directions for Future Research

Our study has some limitations that provide directions for future research. First, although this study collected questionnaires at different time points that were completed anonymously, the cross-sectional design and questionnaires were based on self-reported responses by the participants, so the results were inevitably influenced by common method bias. Future research should adopt a longitudinal tracking design or an experimental design to better identify the causal relationship between transformational leadership and perceived overqualification. In addition, we conducted the study in three waves and combined the paper-and pencil version and online version. Due to the uncertainty of employee access to the online version, additional samples will be missing. Hence, different questionnaires should be designed for different variables and completed by different participants to further eliminate common method variance in the research results.

transformational leadership scale, which failed to fully reflect the differences in employees’ subjective perceptions of transformational leadership. Therefore, future research should try to collect data through experiments or subjective measures to verify the accuracy and reliability of the model.

Finally, the hypotheses were tested with only 351 samples in two industries, so the sample size and the number of industries were relatively small; therefore, the external validity of our hypotheses should be further tested. Future research should further examine the effect of transformational leadership on employees’ psychology, behavior, and performance with a larger study sample.

### Conclusion

The phenomenon that employees feel overqualified seems to be increasingly prevalent around the world. The studies we conducted revealed that transformational leadership can reduce employees’ perceived overqualification by increasing their career growth opportunities, in addition, supervisor–subordinate guanxi can moderate the mediated relationship. Our research also provides the basis for practical recommendations for reducing the individual’s perceived overqualification.

## Data Availability Statement

The original contributions presented in the study are included in the article/Supplementary Material, further inquiries can be directed to the corresponding author.

## Ethics Statement

The studies involving human participants were reviewed and approved by the Shanghai Zhenhua Port Machinery Company and Ford Motor (China) Company Ethics Committee. The participants provided their written informed consent to participate in this study. Written informed consent was obtained from the individual(s) for the publication of any potentially identifiable images or data included in this article. Both employees were given the opportunity to refuse to participate, to omit questions or to withdraw from the study at any time without penalization.

## Author Contributions

All authors listed have made a substantial, direct and intellectual contribution to the work, and approved it for publication.

## Conflict of Interest

The authors declare that the research was conducted in the absence of any commercial or financial relationships that could be construed as a potential conflict of interest.

## Footnotes

1 https://www.wjx.cn
